# Plasma membrane glycosphingolipid signaling: a turning point

**DOI:** 10.1007/s10719-021-10008-w

**Published:** 2021-08-16

**Authors:** Elena Chiricozzi

**Affiliations:** Department of Medical Biotechnology and Translational Medicine, University of Milano, Milano, Italy

**Keywords:** Glycosphingolipid, Ceramide, GM1 oligosaccharide, Plasma membrane interaction, IGO award, Early career award

## Abstract

Plasma membrane interaction is highly recognized as an essential step to start the intracellular events in response to extracellular stimuli. The ways in which these interactions take place are less clear and detailed. Over the last decade my research has focused on developing the understanding of the glycosphingolipids-protein interaction that occurs at cell surface. By using chemical synthesis and biochemical approaches we have characterized some fundamental interactions that are key events both in the immune response and in the maintenance of neuronal homeostasis. In particular, for the first time it has been demonstrated that a glycolipid, present on the outer side of the membrane, the long-chain lactosylceramide, is able to directly modulate a cytosolic protein. But the real conceptual change was the demonstration that the GM1 oligosaccharide chain is able, alone, to replicate numerous functions of GM1 ganglioside and to directly interact with plasma membrane receptors by activating specific cellular signaling. In this conceptual shift, the development and application of multidisciplinary techniques in the field of biochemistry, from chemical synthesis to bioinformatic analysis, as well as discussions with several national and international colleagues have played a key role.

## Introduction

It is a great honour and privilege to be this year’s recipient of the International Glycoconjugate Organization (IGO) Young Glycoscientist Award.

As a biochemist, my research focuses on lipid biochemistry and in particular characterising the sphingolipids involvement in the regulation of the cell homeostasis [2–4]. Although my work in this area only began during my graduate studies in 2010, I have been amazed to be, together with my research team, the author of important discoveries in the field of membrane interactions between proteins and lipids in the last decade. These advancements have been the result of multidisciplinary collaboration in both domestic and international research institutes: the broad collaborative network I sowed along my career represents for my research a lifeline.

My key contributions in this research field have been the application of approaches based both on the molecular study thanks to proteomics and bioinformatics techniques, and the application of chemical synthesis products, such as photoactivable compounds, in which, historically, my research group is on top of the world. Using these approaches, we have greatly improved our understanding of the mechanisms by which membrane lipids interact with specific proteins to ensure adequate cell signalling.

Therefore, in this summary of my contributions I will try to highlight these important and new discoveries so that they may be the key to opening a new perspective on lipid-mediated signalling across the plasma membrane.

## Glycosphingolipids chemistry and their biochemical role

**Fig. 1:**
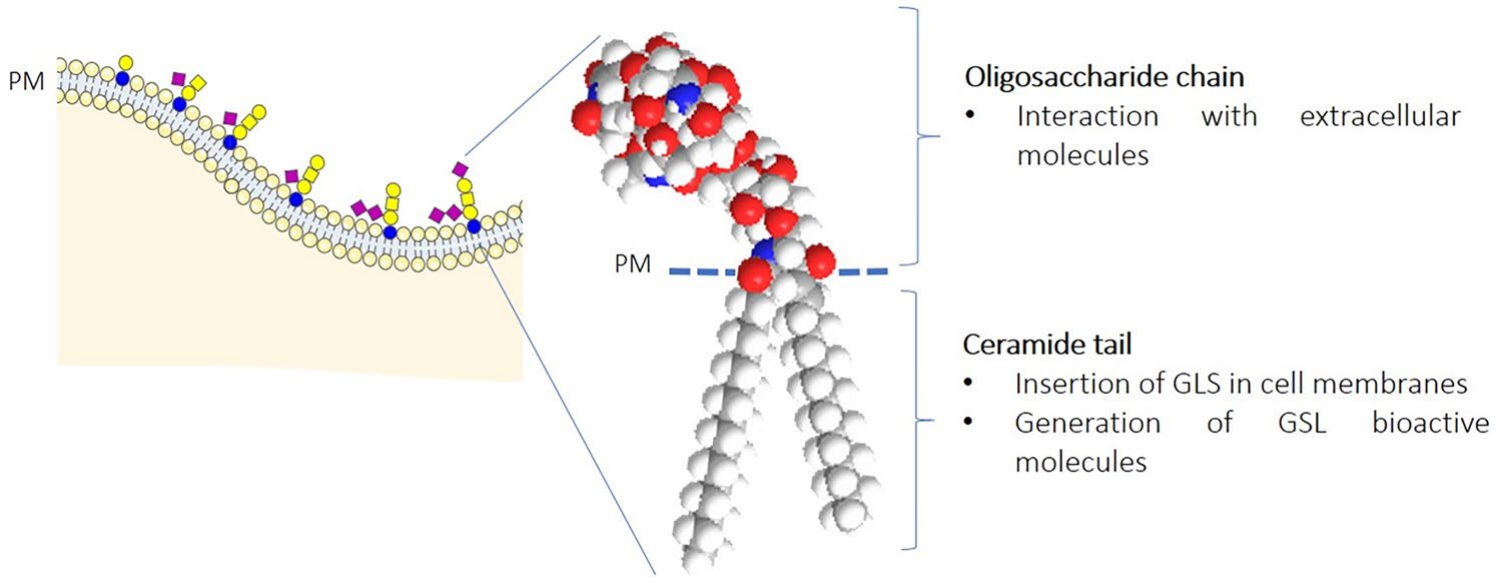
Plasma membrane glycosphingolipid structure: the oligosaccharide head and the ceramide tail. PM: plasma membrane. Glycosphingolipid sugar code is according to Varki *et al.* [49]

Glycosphingolipids are a family of membrane lipids with important roles in the regulation of the fluidity and subdomain structure of the lipid bilayer characterized by particular physical-chemical properties that allow to act as organizers of cell membrane [5–8]. These molecules are strongly amphiphilic: they are characterized by a hydrophobic portion, embedded in the lipidic core of biological membranes, and by a hydrophilic portion protruding in the extracellular milieu (Fig. 1).

The *ceramide* backbone confers some physical-chemical properties to the sphingolipids that differ from those of other membrane lipids. In fact, the amidic linkage, with the contemporary presence in the same molecule of a hydrogen bond donor and an acceptor group (the carbonyl oxygen and the amidic hydrogen), allows the formation of a thick network of hydrogen bonds among different sphingolipid molecules in the lipid bilayer. The presence of hydrogen linkages considerably stabilizes the sphingolipid segregation in specific membrane areas, which appear enriched with this lipid family and for this reason they have been defined “glycosphingolipid-rich membrane domains” [9–12]. In addition, the numerous hydrogen bonds in these domains confer rigidity and resistance to the membrane allowing their differentiation by physical-chemical properties from the remaining membrane.

On the other side of the molecule there is the hydrophilic head that faces the extracellular environment: the *saccharide core*. This is small and composed by one or two saccharides in neutral sphingolipids, while it is composite and more complex in gangliosides, glycosylated sphingolipids characterized by the presence of sialic acid. For its part, the oligosaccharide head can interact through mild hydrophilic bonds, *i.e*., hydrogen bonds, with neighbouring membrane components or extracellular ligands.

Recent results suggest new aspects of lipid function: not only structural components of cell membrane but also fundamental actors of signalling and regulatory pathways. Unexpectedly, advances in biochemical and molecular studies of glycosphyngolipid functions during the past 3 decades, revealed that biological active glycosphyngolipids (ceramide, ceramide 1 phosphate, glucosil-ceramide, lactosylceramide, galactosylceramide, sphingosine, sphingosine 1 phosphate, ganglioside) have key roles in the regulation of several fundamental biological processes: they act also as effector molecules and not only as inert precursor and products of glycosphyngolipids metabolism [13–21].

In fact, once understood the conformation of these molecules, it’s easy to image how the monomeric glycosphingolipids can interact, through the lipid component and by the oligosaccharide one, both with the membrane components (*i.e*., proteins and lipids), segregating and clustering into the membrane and with extracellular actors. Therefore, these molecules forming cell type specific profiles have essential roles in several aspects of cell homeostasis: cell growth, differentiation, morphogenesis, cell to matrix interaction, cell to cell communication and so on.

Below I report two of my researches that highlight the importance of both the *ceramide tail* and the *saccharide head* of these molecules as a starting point for the modulation of the plasma membrane signalling.

## Ceramide tail role in the formation of Lactosylceramide lipid rafts in neutrophil cells

The biochemical study on glycosphingolipid-mediated signalling started in collaboration with the laboratory of Professor Kazuisa Iwabuchi, at the Juntendo University of Tokyo, where I spent 9 months carrying out a project aimed at understanding the role of a specific glycosphingolipid called Lactosylceramide (LacCer) in modulating the cell signalling involved in neutrophils response upon bacteria’s infection [22].

The innate immune system is the first line of defence against pathogenic microorganisms, such as bacteria, fungi, and viruses. Phagocytes, such as neutrophils and macrophages, play an important role in the innate immune system by recognizing, engulfing, and eliminating pathogens. It has been suggested that lipid membrane microdomains of phagocytes are involved in these innate immune responses, including superoxide generation, cell migration, and phagocytosis. Glycosphingolipids are highly enriched in specialized membrane microdomains where they participate to the process of transduction of information across the membrane. Recent proteomic analyses of microdomains from phagocytes have provided insight into membrane microdomain-mediated functions in the processes of phagocytosis [23–26]. LacCer, a neutral glycosphingolipid, is abundantly expressed on human neutrophils and, specifically, recognizes several pathogenic microorganisms [27, 28]. LacCer forms membrane microdomains coupled with the Src family kinase Lyn on the plasma membrane, and ligand binding to LacCer activates Lyn, resulting in neutrophil functions, such as superoxide generation and migration [22, 29, 30]. In contrast, neutrophilic-differentiated HL-60 cells, highly used in basic research instead of human neutrophile, do not have Lyn-associated LacCer-enriched microdomains and lack LacCer-mediated functions. In neutrophil plasma membranes, the very long fatty acid C24:0 and C24:1 chains are the main components of LacCer, whereas plasma membrane of D-HL-60 cells mainly includes C16-LacCer species. LacCer species containing very long fatty acid chains are thus indispensable for the association of Lyn with LacCer-enriched microdomains and LacCer-mediated functions [22, 29, 30].

**Fig. 2:**
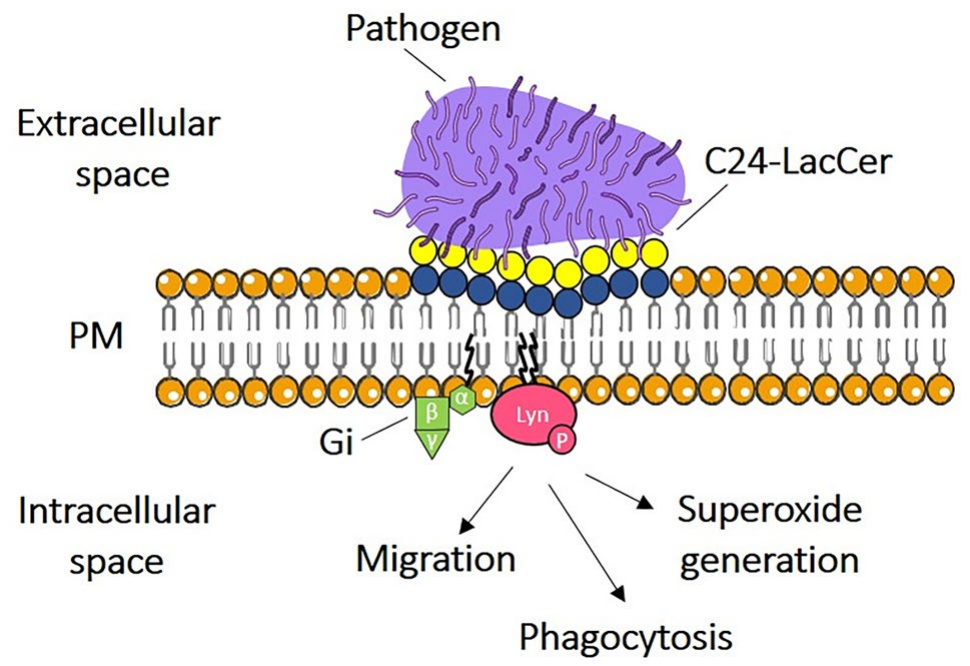
Proposed mechanism diagram for LacCer-lipid raft mediated function in inflammatory response. C24-LacCer directly associates with the cytoplasmic protein Lyn via myristic/palmitic chains promoting its phosphorylation and activation of other proteins (Gi family protein) within plasma membranes lipid rafts of neutrophil cells. This specific C:24-LacCer/Lyn interaction finally leads to phagocytosis, superoxide generation and cell migration following CD11b/CD18 integrin translocation in C:24-LacCer microdomain in response to bacterial infection. Modified from [22]. Glycosphingolipid sugar code is according to Varki *et al*. [49]

With this project we want to study the role of very long fatty acid-LacCer species in the physical and functional coupling with Lyn and in microdomain-mediated functions in the processes of phagocytosis to determine the molecular mechanisms underlying these functions. For this purpose, our chemical group developed the tritium-labelled and photoactivale LacCer derivatives with the aim to give a name to these proteins. To understand what happens at the level of plasma membrane microdomain, we prepared the plasma membrane microdomains to analyse and to recognize the protein associated to the LacCer environment. By means of cross linking and immunoprecipitation experiments with photoactivable and radioactive [^3^H]-C24/C16-(N_3_)-LacCer we showed that Lyn and LacCer with long acyl chain have a direct interaction in LacCer-enriched membrane domain from neutrophilic cells (Fig. 2). First biological results with analogues are encouraging. In fact, they seem to confirm the initial hypothesis related to the requirement of long chains LacCer for the innate responses in human neutrophils.

Our results prove that glycosphyngolipids with a long fatty acid chain of Ceramide moiety, in our specific case LacCer, could be the key-player on the signal transduction across the cellular plasma membrane, modulating membrane interdigitation, reducing the membrane thickness and forming a specific plasma membrane microdomains. We know that, for LacCer mediated, Lyn activation is necessary: LacCer molecular species with ceramide contain a very long fatty acid chain, but also β-Gal(1-4)-β-Glc disaccharide structure of LacCer. This means that, in turn, the oligosaccharide chain is able to recognize some specific proteins belonging to the extracellular space. The synthesis of new LacCer photoactivable analogues will certainly help in the identification of these new players opening a new research field.

## Oligosaccharide role in the GM1 ganglioside neuronal functions

Since 2015, my research activity has mainly been focused on GM1 ganglioside in relation to both neuronal physiological and pathological implication [31–41]. Together with my research group, I give a very important contribution to the understanding of the role of GM1 oligosaccharide as the bioactive portion of the GM1 molecule, opening a new prospective on the ganglioside-mediated signalling across the plasma membrane. Furthermore, the identification of the GM1 oligosaccharide as a membrane-permeable GM1 analogue with superior ability to access intraneuronal GM1 functional sites may hold considerable therapeutic promises for sporadic Parkinson’s disease (PD). Like all the important discoveries, it derived from the randomness of the moment while we were carrying out a new research on the synthesis of GM1.

*In vitro* and *in vivo* studies have indirectly suggested us that the oligosaccharide portion of the GM1 (OligoGM1) could provide an important contribution. In 1988, a pioneering paper by Shengrund and Prouty observed that the OligoGM1 promoted neuritogenesis [42]. In 2012, Ledeen and coworkers identified LIGA20, a membrane brain permeable analogue of GM1, as an effective (albeit toxic in the long-term) alternative to GM1 [43]. Treatment of GM1-deficient parkinsonian mice with LIGA20 induced beneficial results, including the reduction of *substantia nigra* α-synuclein aggregates. The most important finding of this study was the identification of a hydrophilic GM1 derivative, modified on the ceramide moiety with a dichloroacetyl group instead of the acyl chain, but keeping the entire oligosaccharide intact, which still maintained trophic potential. This suggested that the ceramide structure is not critically related to GM1 modulatory effects. In 2015, Scheneider noted that the plasma membrane GM1 increased by intraventricular injection of *Vibrio cholerae* sialidase exerted a neuroprotective effect on the damaged nigrostriatal dopaminergic system of MPTP mice [44]. This enzyme removes sialic acid residues from brain polysialogangliosides (*i.e*., GD1a), thus increasing plasma membrane GM1, further evidence that the oligosaccharide may act as the mediator of GM1 function.

Accordingly, my collaborators and I recently reported that the soluble OligoGM1 (II^3^Neu5Ac-Gg_4_) replicates the neurotrophic and neuroprotective properties of the entire GM1 molecule: GM1 exerts its bioactivity through its hydrophilic head, which protrudes into the extracellular environment and therefore acts at the cell surface by interacting with plasma membrane proteins [31–41].

**Fig. 3:**
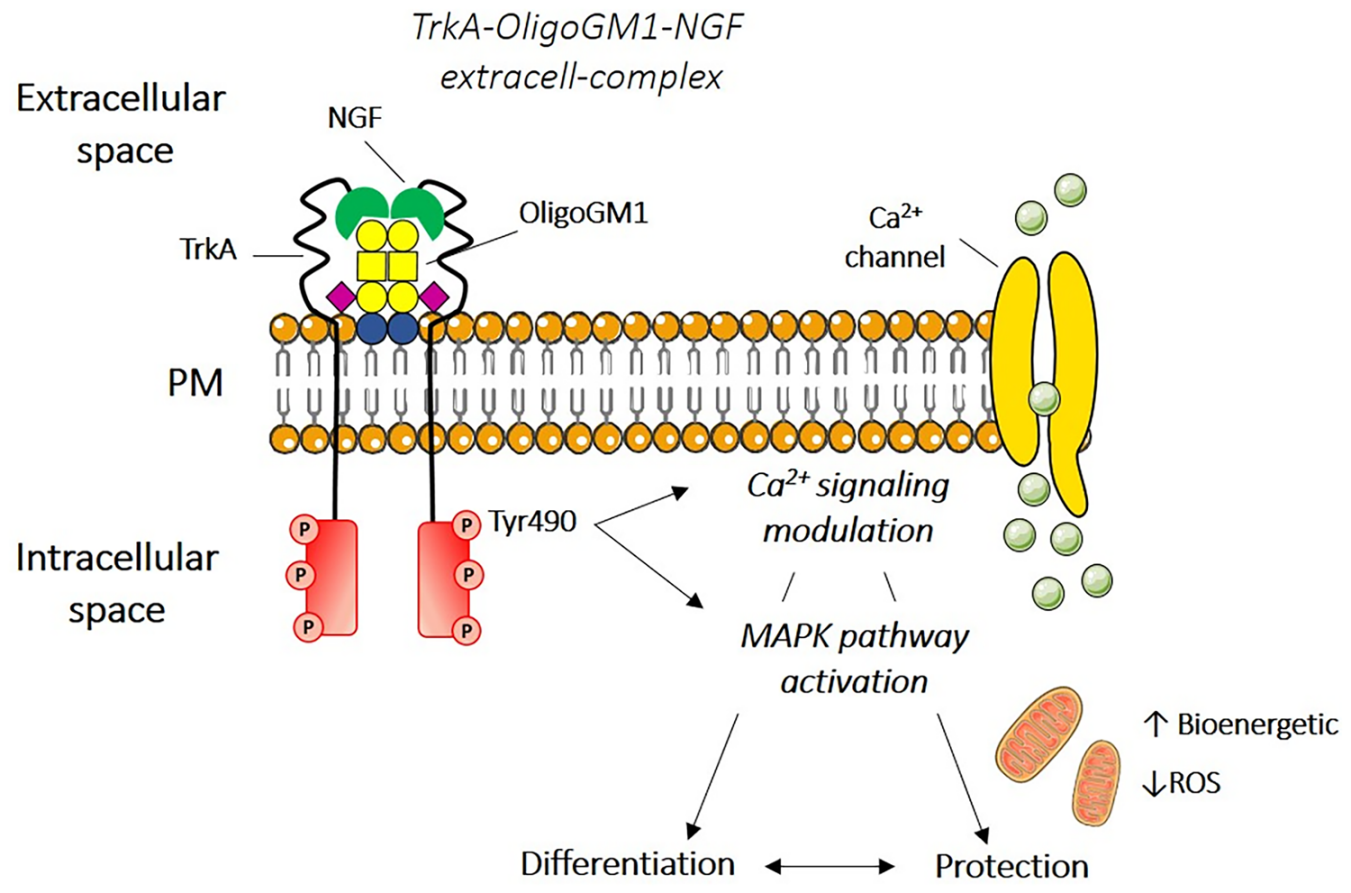
Proposed mechanism diagram of the GM1 oligosaccharide mediated functions. GM1 ganglioside through its oligosaccharide chain stabilizes the TrkA-NGF complex on the cell surface triggering the phosphorylation of TrkA on tyrosine 490 (Tyr490) promoting MAPK signaling. This induces the activation of multiple intracellular pathways that finally lead to neuronal differentiation, protection and restoration. Modified from [31, 33, 37, 40]. Glycosphingolipid sugar code is according to Varki *et al.* [49]

In fact, I reported in neuronal cells that, within the entire GM1 molecule, OligoGM1 is the actual moiety responsible for GM1 neurotrophic properties [31–41]. OligoGM1 directly interacts with the NGF receptor TrkA, leading to MAPK (*i.e*. ERK1/2) downstream pathway activation [31, 33, 38], to cell differentiation [31, 38] and to sustain MPTP neuroprotection [32] through mitochondria bioenergetic [32, 37] and calcium modulation [32, 40] (Fig. 3). Following its injection in wild-type mice, OligoGM1 was found to reach brain areas [34], and by using an *in vitro* human blood brain barrier (BBB) model, OligoGM1 showed a 20-fold higher crossing rate than that of entire GM1 and crossed the BBB directly by a passive-paracellular mechanism [36]. Importantly, OligoGM1 systemically administered to a PD mouse model (the GM1 deficient *B4galnt1*^+/-^ one) was found to completely rescue the physical symptoms, to reduce αS aggregates, to restore tyrosine hydroxylase neurons and to recover the neurotransmitters’ levels [34]. Altogether, this evidence confirms that the specific role of ganglioside GM1 in neuronal homeostasis is mediated by its oligosaccharide: this bioactive portion, protruding in the extracellular environment, acts at the cell surface by a direct interaction with specific proteins.

From this premise, it emerged our idea that the plasma membrane OligoGM1 decline could be the trigger for sustained pathway impairment as the etiopathogenetic target event of sporadic PD, which is characterized by a systemic reduction of GM1. Supported by extensive research into the physiological function and pathological implications of ganglioside GM1 [39, 45–48] and by solid data on OligoGM1 functions [31–40], I propose a new key to untying the knot of complex neurodegenerative diseases: the OligoGM1 as an entirely new neurotrophic player, able to counteract all the multifactorial aspects of PD. Indeed, the soluble OligoGM1 loses the amphiphilicity of the entire ganglioside and gains the possibility to efficiently cross the BBB, reaching the target neurons. Here, without entering the cells but interacting with cell surface proteins, OligoGM1 should be enough to restore and maintain the correct neuronal homeostasis, through the activation of trophic plasma membrane signal and alpha-synuclein clearance, with significant therapeutic implications. The prompt restoration of all the mechanisms (*i.e*., oxidative stress and mitochondrial dysfunction, inflammation, excitotoxicity, loss of trophic support and protein accumulation/aggregation) that are likely to contribute and to exacerbate the pathogenesis of Parkinson and in general to multifactorial neurodegenerative disorders (*i.e*., Alzheimer’s disease) is fundamental to the recovery of injured cells and tissues, particularly those with delicate equilibria (*i.e*., dopaminergic neurons). Otherwise, OligoGM1 should drive plasma membrane trophic signalling inducing a reduction in glia/microglia activation, oxidative stress, mitochondria dysfunction, excitotoxicity and α-synuclein aggregation resulting in the recovery of damaged neurons and the restoration of normal healthy phenotype [39]. These concepts bring out the OligoGM1 as a cutting-edge molecule able to recover and cope with all aspects of neurodegenerative diseases, for which, to date, there are no effective therapies.

Overall, my aim is to provide a significant advance in the understanding of the molecular mechanism by which GM1, and in particular its oligosaccharide portion, is responsible for the onset occurring in sporadic PD. I expect to provide novel insights into the GM1-Parkinson pathogenesis, as well as into the mechanism occurring at the plasma membrane level that might account for the reduced neuroactivity in Parkinson neurons, but also in the extra-central tissues.

These findings will open a new perspective on the ganglioside mediated signalling across the plasma membrane. Furthermore, the identification of a novel membrane-permeable GM1 analogue with superior ability to access intraneuronal GM1 functional sites holds considerable therapeutic promises.

## What’s next

The field of glycobiology and, in particular, the studies concerning the molecular mechanisms underlying the interaction between membrane lipids and proteins are fields of research that represent the starting engine of everything that happens inside our cells in response to external stimuli. Even if it seems that everything has already been discovered, characterizing in a fine and more detailed way, with the new technical approaches available today, will allow unexpected discoveries which may have even more unexpected applications.

A key challenge within this field will continue to be the identification of previously unknown interactions such as the one of GM1 oligosaccharide. The goal of my research group is to see OligoGM1 applied to the clinic since its ability to recover the neuro-degenerated Parkinson's phenotype is truly sensational and at the same time to develop a tool for the large-scale synthesis of OligoGM1. The hope is to verify how this molecule can be useful for all other diseases where there is a deficiency of GM1 or where GM1 has been used in the past but with poor results due to the impossibility of GM1 to overcome an intact BBB.

## Ganglioside nomenclature is in accordance with IUPAC-IUBB recommendations [1]

BBB: blood brain barrier
GM1: II^3^Neu5Ac-Gg_4_Cer, β-Gal-(1-3)-β-GalNAc-(1-4)-[α-Neu5Ac-(2-3)]-β-Gal-(1-4)-β-Glc-Cer
LacCer: Lactosylceramide, β-Gal(1-4)-β-Glc-Cer
MPTP: 1-Methyl-4-phenyl-1,2,3,6-tetrahydropyridine hydrochloride
NGF: nerve growth factor
OligoGM1: GM1 oligosaccharide, II^3^Neu5Ac-Gg_4_
PD: Parkinson’s disease
Trk: neurotrophin tyrosin kinase receptor
